# Sequential somatic mutations upon secondary anti-HER2 treatment resistance in metastatic ERBB2^S310F^ mutated extramammary Paget’s disease

**DOI:** 10.18632/oncotarget.27272

**Published:** 2019-11-19

**Authors:** Thierry M. Nordmann, Olivia Messerli-Odermatt, Larissa Meier, Sara Micaletto, Thomas Coppetti, Mirjam Nägeli, Jivko Kamarachev, Ken Kudura, Sandra N. Freiberger, Tamara Rordorf, Joanna Mangana, Ralph Braun, Reinhard Dummer

**Affiliations:** ^1^Department of Dermatology, University Hospital Zurich, Zurich, Switzerland; ^2^Department of Nuclear Medicine, University Hospital Zurich, Zurich, Switzerland; ^3^Department of Pathology and Molecular Pathology, University Hospital Zurich, Zurich, Switzerland; ^4^Department of Oncology, University Hospital Zurich, Zurich, Switzerland

**Keywords:** ERBB2 protein, Paget disease, extramammary, trastuzumab, lapatinib

## Abstract

Metastatic extramammary Paget’s disease is a rare adenocarcinoma with poor prognosis. Several reports of human epidermal growth factor receptor 2 alterations point to its pathogenic role in the disease. However, the occurrence of treatment resistance to anti-HER2 therapy demand the need for further knowledge. We report of a patient with metastatic penoscrotal extramammary Paget’s disease, with an *ERBB2^S310F^* mutation, in which near complete response was achieved upon treatment with trastuzumab and carboplatin. However, after 10 cycles of trastuzumab and carboplatin, widespread metastasis re-occurred. Analysis of a newly developing metastasis revealed additional genomic alterations including ERBB3^A232V^ and *PIK3CA^G106V^* point mutations as well as *MET* and *CDK6* amplification, providing a potential mechanism of acquired treatment resistance. Therefore, *ERBB* family inhibitor afatinib was initiated. Unfortunately, the patient succumbed to disease-related complications shortly after treatment initiation. This is the first report of *ERBB2^S310F^* mutated, metastatic extramammary Paget’s disease with secondary resistance to trastuzumab / carboplatin, potentially due to additional acquired genomic alterations. This case contributes to the growing evidence of HER2 in the pathogenesis of metastatic extramammary Paget’s disease and emphasizes the importance of repetitive, genomic analysis in rare diseases.

## INTRODUCTION

Extramammary Paget’s disease (EMPD) is a rare intraepithelial adenocarcinoma, localized in apocrine gland-rich sites, most commonly perianal, vulvar and penoscrotal. The clinical presentation is discreet and eczematous in nature, often causing a delay in diagnosis. Most cases of EMPD are in-situ and have an excellent prognosis. In 5-25%, dermal invasion is visible, correlating with a poor prognosis. Due to the rarity of the disease, evidence-based management in both in-situ and metastatic disease is scarce. In the past, various combinatorial chemotherapeutic regimens were applied, with limited success. Recently, growing evidence suggests a central role of human epidermal growth factor receptor (HER) 2 in the disease. Yet incomplete responsiveness and the occurrence of treatment resistance to anti-HER2 treatment frequently occurs.

## RESULTS

**Figure 1:**
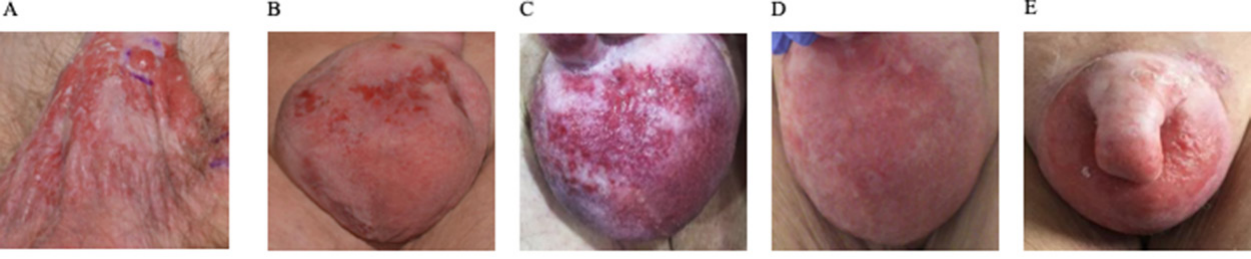
Therapeutic efficacy assessed by clinical penoscrotal examination. Imaging of the penoscrotal region **(A)** at the initial presentation, **(B)** following recurrent disease after radiation therapy **(C)** after lapatinib treatment **(D)** best response during trastuzumab and carboplatin treatment, and **(E)** disease progression after 10 cycles of trastuzumab and carboplatin.

**Figure 2:**
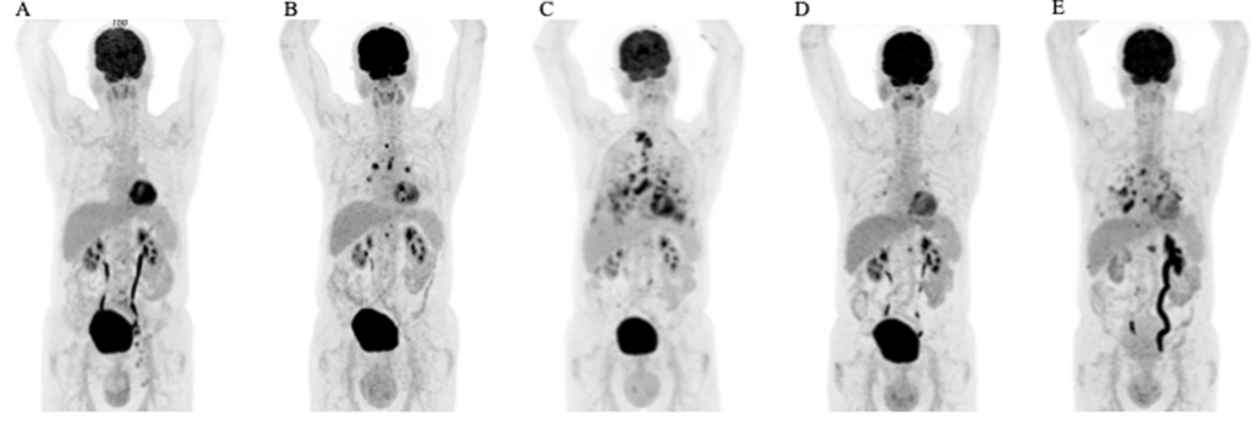
Therapeutic efficacy assessed by FDG-PET/CT scans. Coronal maximum intensity projection images **(A)** at the initial presentation, **(B)** following recurrent disease after radiation therapy **(C)** after lapatinib treatment **(D)** best response during trastuzumab and carboplatin treatment, and **(E)** disease progression after 10 cycles of trastuzumab and carboplatin.

We report on an 80-year-old patient in excellent general condition (ECOG 0), who was referred to our clinic for further treatment of a chronic scrotal eczema. Upon physical examination, erythematous plaque with multiple nodules affecting the skin of the scrotum was seen, along with prominent inguinal lymphadenopathy (Figure 1A). Histologic analysis showed an ulcerated and invasive adenocarcinoma with Paget cells, consistent with the diagnosis of extramammary Paget’s disease. Furthermore, enlarged and metabolic active inguinal, iliacal and interaortocaval lymph-nodes were detectable on PET/CT-imaging (Figure 2A). Indeed, fine needle aspiration (FNA) confirmed the presence of lymph node metastasis. Fluorescence in situ (FISH) for HER2 amplification was negative and immune-histochemical staining for HER2 was 2+, classifying the patient as HER2 negative, according to current ASCO/CAP Guidelines [1]. Urological and gastroenterological examination did not reveal underlying malignancy of the prostate or gastrointestinal tract, respectively. After interdisciplinary discussion, the patient was opted for radiotherapy of the penoscrotal region and metabolic active lymph nodes (cumulative 60Gy), which was well tolerated.

6 months into follow-up, scrotal inflammation re-occurred (Figure 1B). Multiple mapping skin biopsies revealed apocrine adenocarcinoma in situ. PET/CT imaging showed new metabolic activity of the thoracal and retrocrural lymph-nodes, and a metabolically active nodulous in the superior right lobe of the lung (Figure 2B). FNA of the pulmonal nodule confirmed the presence of pulmonary metastasis. In order to identify targetable molecular alterations, next-generation sequencing (Oncomine Focus Assay) of the scrotal tumor was performed, revealing an activating *ERBB2^S310F^* point mutation. Accordingly lapatinib treatment (1250mg daily) was initiated [2]. However, the patient’s general condition deteriorated continuously along with respiratory insufficiency, leading to the necessity of continuous oxygen supplementation. While scrotal inflammation worsened, PET/CT imaging showed disease progression with pleural effusions and bone metastasis (Figure 1C and 2C, respectively). Again, histological analysis of FDG-avid bronchial mucosa was performed, confirming the presence of Paget-cells. Interestingly, HER2 immune-histochemical staining was strongly positive (3+) classifying the patient as HER2 positive. Therefore, lapatinib was discontinued and the patient was treated with trastuzumab (6mg/kg body weight) and carboplatin (300mg) every 3 weeks. Within one month, the patient’s general condition improved rapidly and oxygen supplementation was not further needed. In addition, scrotal inflammation resolved (Figure 1D). PET/CT imaging showed partial response of all lymph node metastasis, as well as subtotal response of pulmonary and bone metastasis (Figure 2D). A single bone metastasis at the cervical vertebra was irradiated due to local disease progression.

After 10 cycles of trastuzumab and carboplatin, follow-up PET/CT imaging displayed disease progression, with re-occurrence of multiple lymph-node and pulmonary metastasis (Figure 2E). Scrotal inflammation was mildly increased (Figure 1E). Molecular analysis (Oncomine Focus Assay) from a newly developing and metabolic active hilar lymph node metastasis was performed. Apart from the known *ERBB2^S310F^* mutation, additional genomic alterations were identified, including *ERBB3^A232V^* and *PIK3CA^G106V^* point mutations, and amplification of *CDK6*. In order to identify further potential treatable targets, Foundation One analysis was performed, revealing additional, equivocal *MET* amplification. Given the fact, that ERRB3 mutations have been associated with resistance to *ERBB2* targeted treatment strategies, trastuzumab / carboplatin was discontinued and afatinib, an *ERRB* family Inhibitor, was initiated [3]. Unfortunately, 8 days after initiation of afatinib, the patient died of community acquired pneumonia.

## DISCUSSION

Advances have been made in the treatment of metastatic extramammary Paget’s disease. The identification of *ERBB2* amplifications and somatic mutations in EMPD has enabled disease specific, targeted treatment [4]. Indeed, 15-80% of all EMPD patients show immune-histochemical HER2 positivity, associated with a biologically aggressive phenotype [5]. Accordingly, anecdotal anti-HER2 treatment has significantly improved the outcome of metastasized EMPD, emphasizing its pathogenic role the disease. However, incomplete responsiveness and the occurrence of treatment resistance to anti-HER2 therapies demand the need for further knowledge.

The tyrosine receptor kinase HER2 causes increased MAPK/ERK and PI3K/mTOR pathway signaling, accelerating cell growth and survival. Historically, HER2 status is assessed by immuno-histochemistry (IHC) and fluorescence in-situ hybridization (FISH) for the detection of overexpression and amplification, respectively. However, nonamplified, activating ERBB2-mutations are not detected by IHC / FISH. Furthermore, only 30% of all *ERBB2* alterations are amplifications, and nearly 2% of all tumors carry *ERBB2* mutations [4, 6]. The activating S310F point mutation is the most common somatic mutation in *ERBB2* and has been successfully targeted in EMPD [6, 7]. Given the similar beneficial response to anti-HER2 treatment in amplified and non-amplified *ERBB2* alterations, patients with a potential benefit to anti-HER2 treatment may not be identified using IHC / FISH.

Here we report on the clinical efficacy of trastuzmab / carboplatin in an 80-year-old male patient with metastatic penoscrotal EMPD, harboring a somatic *ERBB2^S310F^* mutation, with primary resistance to lapatinib. Upon disease progression, sequential genetic profiling revealed additional somatic point mutations in *ERBB3 (p.A232V)* and *PIK3CA (p.G106V)* as well as *MET* and *CDK6* amplification. *ERBB3^A232V^* is a missense, hot-spot mutation within the extracellular domain, causing anchorage-independent growth and signaling when HER2 kinase activity is present [3]. Accordingly, in the presence of *ERBB2^S310F^*, additional somatic *ERBB3^A232V^* may have caused a compensatory mechanism of resistance to *ERBB2* targeted therapy. Indeed, *ERBB3* mutations have been shown to cause resistance to *ERBB2* targeted therapy [3]. *PIK3CA^G106V^* is a missense, activating hot-spot mutation within the adaptor-binding domain of the catalytic subunit of the phosphoinositide 3-kinase, stimulating its lipid kinase activity [8]. Activation of downstream signaling pathways are a known mechanism of secondary resistance and mutations in *PIK3CA* are associated with resistance to trastuzumab [9]. In addition, both *MET* and *CDK6* amplification have been described to contribute to trastuzumab resistance in HER2-overexpressing breast cancer [10, 11].

This is the first description of acquired somatic alterations occurring after secondary treatment resistance in a patient with non-amplified, *ERBB2* mutated, metastatic EMPD. Interestingly, these acquired genetic alterations may have caused treatment resistance and contribute to the understanding of commonly occurring secondary treatment failure of anti-HER2 treatments in metastatic EMPD. Furthermore, this case provides rationale for repetitive genomic analysis in treatment resistant EMPD lesions and contributes to the growing evidence of *ERBB2* in the pathogenesis of metastatic extramammary Paget’s disease.

## MATERIALS AND METHODS

DNA from FFPE tumor tissue was isolated using the automated Maxwell isolation system (Promega). Next generation sequencing was performed on formalin-fixed paraffin embedded (FFPE) samples, using the Oncomine Focus Assay (Life Technologies / Thermo Fisher) and the FoundationOne panel. Library preparation and sequencing was performed according to the manufacturers’ manuals.
